# Supplementary material for: SNPC-1.3 is a sex-specific transcription factor that drives male piRNA expression in C. elegans
Source: eLife. 2021 Feb 15;10:e60681. doi: 10.7554/eLife.60681 (PMC7884074; doi:10.7554/eLife.60681)
Supplement: Transparent reporting form [file elife-60681-transrepform.docx]

***eLife’s* transparent reporting form**

We encourage authors to provide detailed information *within their submission* to facilitate the interpretation and replication of experiments. Authors can upload supporting documentation to indicate the use of appropriate reporting guidelines for health-related research (see [EQUATOR Network](http://www.equator-network.org/%20)), life science research (see the [BioSharing Information Resource](https://biosharing.org/)), or the [ARRIVE guidelines](http://www.plosbiology.org/article/info:doi/10.1371/journal.pbio.1000412) for reporting work involving animal research. Where applicable, authors should refer to any relevant reporting standards documents in this form.

If you have any questions, please consult our Journal Policies and/or contact us: [editorial@elifesciences.org](mailto:editorial@elifesciences.org).

**Sample-size estimation**

- You should state whether an appropriate sample size was computed when the study was being designed
- You should state the statistical method of sample size computation and any required assumptions
- If no explicit power analysis was used, you should describe how you decided what sample (replicate) size (number) to use

Please outline where this information can be found within the submission (e.g., sections or figure legends), or explain why this information doesn’t apply to your submission:

No power analysis was performed beforehand to determine sample size.

Sample sizes and statistical methods used for each experiment are described in figure legends and Methods.

**Replicates**

- You should report how often each experiment was performed
- You should include a definition of biological versus technical replication
- The data obtained should be provided and sufficient information should be provided to indicate the number of independent biological and/or technical replicates
- If you encountered any outliers, you should describe how these were handled
- Criteria for exclusion/inclusion of data should be clearly stated
- High-throughput sequence data should be uploaded before submission, with a private link for reviewers provided (these are available from both GEO and ArrayExpress)

Please outline where this information can be found within the submission (e.g., sections or figure legends), or explain why this information doesn’t apply to your submission:

Mass spectrometry and ChIP-seq were performed in biological duplicates.

RNA-seq was performed in biological triplicate.

Between two and four biological replicates were performed for western blots and RT-qPCR experiments. Two technical replicates were performed per biological replicate for each RT-qPCR experiment, and the number of technical replicates is noted in the figure legends.

Three to four biological replicates were performed for all fertility and mating experiments.

Biological replicates consisted of independent populations of worms that were grown and processed on separate days from the other biological replicate.

Technical replicates consisted of sampling the same biological replicate multiple times to take into consideration variables such as user, pipetting error, and other external factors that may influence sampling.

The number of biological replicates performed for each experiment is noted in the Methods section.

All mass spectrometry and sequencing data were deposited under the GEO Accession number GSE152831. This information is also noted in the Methods section.

**Statistical reporting**

- Statistical analysis methods should be described and justified
- Raw data should be presented in figures whenever informative to do so (typically when N per group is less than 10)
- For each experiment, you should identify the statistical tests used, exact values of N, definitions of center, methods of multiple test correction, and dispersion and precision measures (e.g., mean, median, SD, SEM, confidence intervals; and, for the major substantive results, a measure of effect size (e.g., Pearson's r, Cohen's d)
- Report exact p-values wherever possible alongside the summary statistics and 95% confidence intervals. These should be reported for all key questions and not only when the p-value is less than 0.05.

Please outline where this information can be found within the submission (e.g., sections or figure legends), or explain why this information doesn’t apply to your submission:

The number of samples (N) per group, definition of center, and type of dispersion measure is provided in the figure legends, labelled in the figures, or in the Methods section. The statistical analysis used is provided in the figure legends.

(For large datasets, or papers with a very large number of statistical tests, you may upload a single table file with tests, Ns, etc., with reference to sections in the manuscript.)

**Group allocation**

- Indicate how samples were allocated into experimental groups (in the case of clinical studies, please specify allocation to treatment method); if randomization was used, please also state if restricted randomization was applied
- Indicate if masking was used during group allocation, data collection and/or data analysis

Please outline where this information can be found within the submission (e.g., sections or figure legends), or explain why this information doesn’t apply to your submission:

Samples were allocated according to different genotypes, experimental treatments, and/or developmental time points. These groups are clearly labelled in figures and figure legends.

For both SNPC-1.3 and TRA-1 ChIP-seq, published ce11 ENCODE-blacklisted regions ([https://github.com/Boyle-Lab/Blacklist/tree/master/lists](https://slack-redir.net/link?url=https%3A%2F%2Fgithub.com%2FBoyle-Lab%2FBlacklist%2Ftree%2Fmaster%2Flists)) were excluded from downstream analyses subsequent to peak-calling.

**Additional data files (“source data”)**

- We encourage you to upload relevant additional data files, such as numerical data that are represented as a graph in a figure, or as a summary table
- Where provided, these should be in the most useful format, and they can be uploaded as “Source data” files linked to a main figure or table
- Include model definition files including the full list of parameters used
- Include code used for data analysis (e.g., R, MatLab)
- Avoid stating that data files are “available upon request”

Please indicate the figures or tables for which source data files have been provided:

Source data for Figure 2 are provided in Tables S1 and S2.

Source data for Figure 4 are provided in Table S3.

Source data for mass spec are provided in Figure S1B and GSE152831.

Source data files are included for qPCR, fertility assays, and image quantification for pronase activation experiments.

Scripts for RNA and ChIP-sequencing are available at <https://github.com/starostikm/SNPC-1.3>. This is noted in the Methods section.
